# Risk Factors of Prolonged Mechanical Ventilation in Infants With Pierre Robin Sequence After Mandibular Distraction Osteogenesis: A Retrospective Cohort Study

**DOI:** 10.3389/fped.2021.587147

**Published:** 2021-04-12

**Authors:** Na Zhang, Zhe Mao, Yingqiu Cui, Yingyi Xu, Yonghong Tan

**Affiliations:** ^1^Department of Anesthesia and Preoperative Medicine, Guangzhou Women and Children's Medical Center, Guangzhou, China; ^2^Department of Stomatology, Guangzhou Women and Children's Medical Center, Guangzhou, China

**Keywords:** mechanical ventilation, Pierre Robin sequence, infants, mandibular distraction osteogenesis, complications

## Abstract

**Background:** After mandibular distraction osteogenesis (MDO), most infants with Pierre Robin sequence (PRS) require mechanical ventilation to assist their breathing. However, the optimal duration of intubation during early mandibular distraction osteogenesis activation is poorly understood. This retrospective study was carried out to identify perioperative risk factors of prolonged mechanical ventilation in infants undergoing MDO.

**Methods:** A total of 95 infants with PRS underwent MDO at Guangzhou Women and Children's Medical Center between 2016 and 2018, and the clinical records of 74 infants who met the selection criteria were analyzed. Of the 74 infants, 26 (35.1%) underwent prolonged mechanical ventilation, 48 (64.9%) did not. *t*-test, Wilcoxon Sum Rank test or chi-squared test were performed to compare variables that might associate with prolonged mechanical ventilation between the two groups, and then, significant variables identified were included in the multivariate logistic regression model to identify independent variables.

**Results:** Univariate logistic regression analysis revealed that age, preoperative gonial angle, and postoperative pulmonary infection were associated with prolonged mechanical ventilation (all *P* < 0.05). Multivariate logistic regression analysis confirmed that the preoperative gonial angle and postoperative pulmonary infection were independent risk factors of prolonged mechanical ventilation (both *P* < 0.05).

**Conclusions:** Infants with PRS and smaller preoperative gonial angle or postoperative pulmonary infection may be more likely to undergo prolonged mechanical ventilation after MDO. For others, extubation may be attempted within 6 days after MDO.

## Background

Pierre Robin sequence (PRS) is a congenital disease characterized by micrognathia, glossoptosis, and upper airway obstruction. It is often accompanied with cleft palate (1). The incidence of PRS varies from 1:5,000 to 1:85,000 across studies due to differences in study populations and diagnostic criteria (2–5). Many infants with PRS suffer from tongue-based airway obstruction and feeding intolerance due to micrognathia and glossoptosis. In recent years, mandibular distraction osteogenesis (MDO) is increasingly used as a first-line surgical treatment of severe airway obstruction in patients with PRS (6–8). Mechanical ventilation is an important adjuvant therapy following MDO, but some patients require prolonged mechanical ventilation, because distraction was not enough and spontaneous breathing had not recovered which may increase the risk of ventilator-associated pneumonia (VAP), prolong hospital stay, and increase mortality (9). The reported duration of postoperative mechanical ventilation varied greatly in different studies and lasted from 1 to 46 days (10–14). Frawley et al. (12) and Marijnissen et al. (13) found that a minimum of 5 days of mechanical ventilation is most prudent considering patient's age, history of preoperative upper airway obstruction, and known difficult intubation. Zhang et al. (14) reported that attempting extubation at least 5 days postoperatively may improve the likelihood of avoiding complication, and in syndromic patients, an attempt to extubate 6 days after post–MDO may be more appropriate. Therefore, in the present study, a mechanical ventilation lasting longer than 6 days was defined as prolonged mechanical ventilation (PMV).

Currently, risk factors and complications of prolonged mechanical ventilation are not well-studied and the optimal duration of intubation during early mandibular distraction osteogenesis activation is poorly understood. Therefore, this study was conducted to identify risk factors of PMV in infants undergoing MDO.

## Materials and Methods

### Subjects

Patients younger than 1 year with PRS who underwent MDO at Guangzhou Women and Children's Medical Center, Guangzhou, China, between November 2016 and November 2018 were included in this study. Demographic and clinical data, such as microlaryngoscopy and bronchoscopy report, computed tomography (CT) of the maxillofacial skeleton, distraction protocol, timing of extubation trial, and respiratory outcomes data were extracted from the electronic medical records of subjects meeting inclusion criteria. Patients were excluded if he/she was older than 1 year and/or had incomplete medical records. The study protocols were complied with the 1975 Declaration of Helsinki and were approved by the Research Ethics Committee of Guangzhou Women and Children's Medical Center, Guangzhou, China and written consent was obtained from the guardian of every child participant.

The trial was registered in China Clinical Trial Registry and the registration number is ChiCTR1800020384.

### Surgical Methods

A multidisciplinary team with members from departments of dentistry, neonatology, pulmonology, anesthesiology and nursing participated in patient assessment and surgical decision-making. The treatment of PRS began with non-invasive techniques at our center. All Children were treated conservatively first by closely checking their breath in the prone position. If the child's respiratory status remained compromised after positioning alone, a placement of a nasopharyngeal airway (NPA) was performed. If NPA failed to relieve airway obstruction, continuous positive airway pressure (CPAP) was applied. Surgical options were mainly reserved for infants with severe airway obstruction who did not respond to non-invasive interventions aimed at mitigating the need for tracheostomy. It was considered to be severe airway obstruction if there were at least three clusters of desaturations with at least 3 dips below 80% within 24 h (4). After each set of interventions, including positioning, NPA, and CPAP, the patient was monitored with pulse oximetry again. In addition, CT scan and fibrobronchoscopy were also performed to judge the severity of airway obstruction. A bronchoscopy was performed before mandibular distraction to rule out secondary airway anomalies which could hamper MDO. All surgical procedures were performed by the same group of surgeons. Bilateral mandibular osteotomies were performed, and the distraction device was fixed with screws on either side. The distraction device (Zhongbang, Xi'an, Shanxi, China) was placed at the mandibular angle. The traction was directed parallel to the connection line between the mandibular point and mental process. The mandibular angle was distracted at 1.4–2.1 mm for 3 days (0.7–1.4 mm each in the morning and evening) and then 1.40 mm daily (0.70 mm each in the morning and evening) until the patient exhibited a prognathism.

### Clinical Indicators

**Figure 1:**
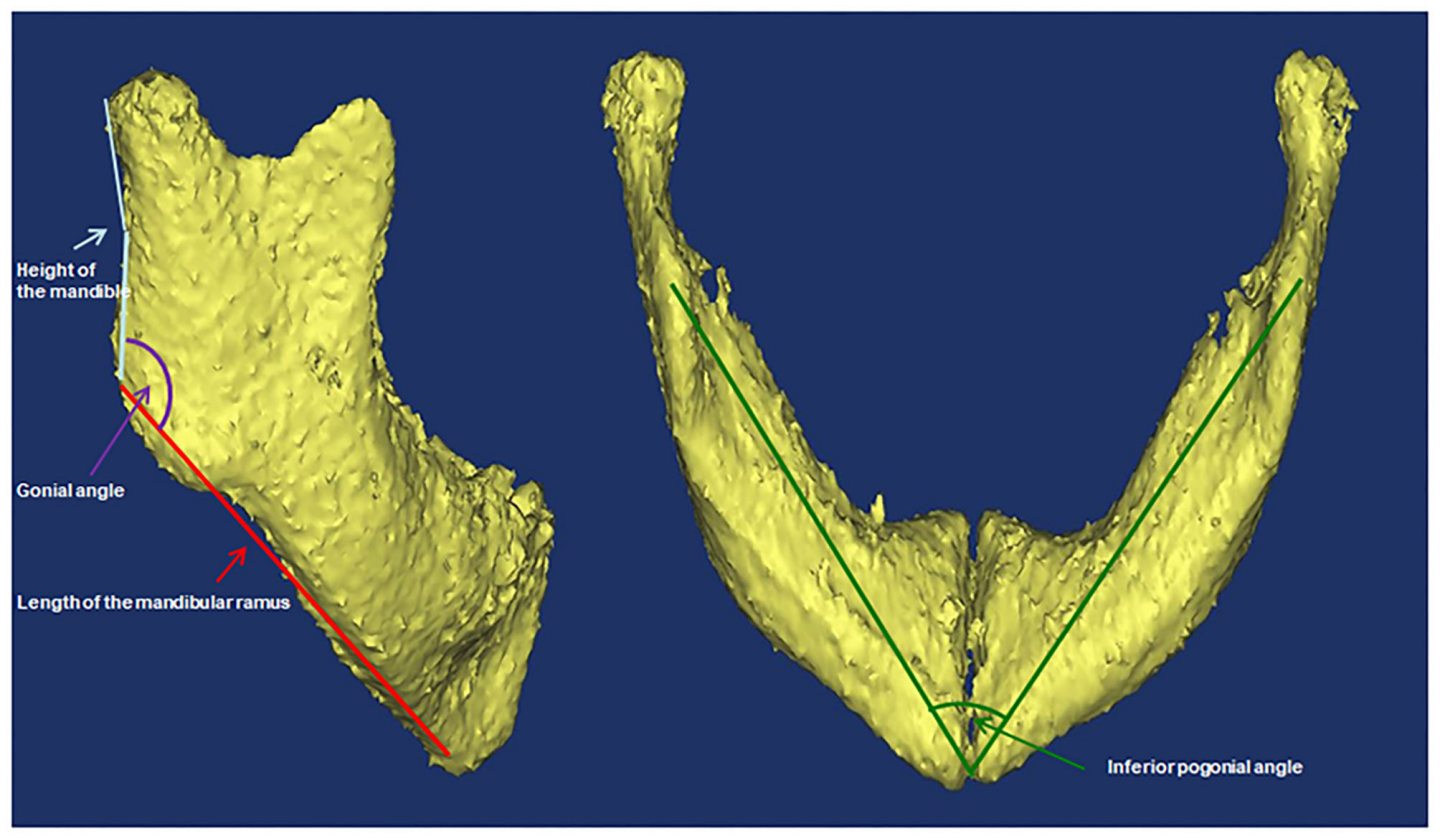
Three-dimensional computed tomographic measurements of the mandible.

**Figure 2:**
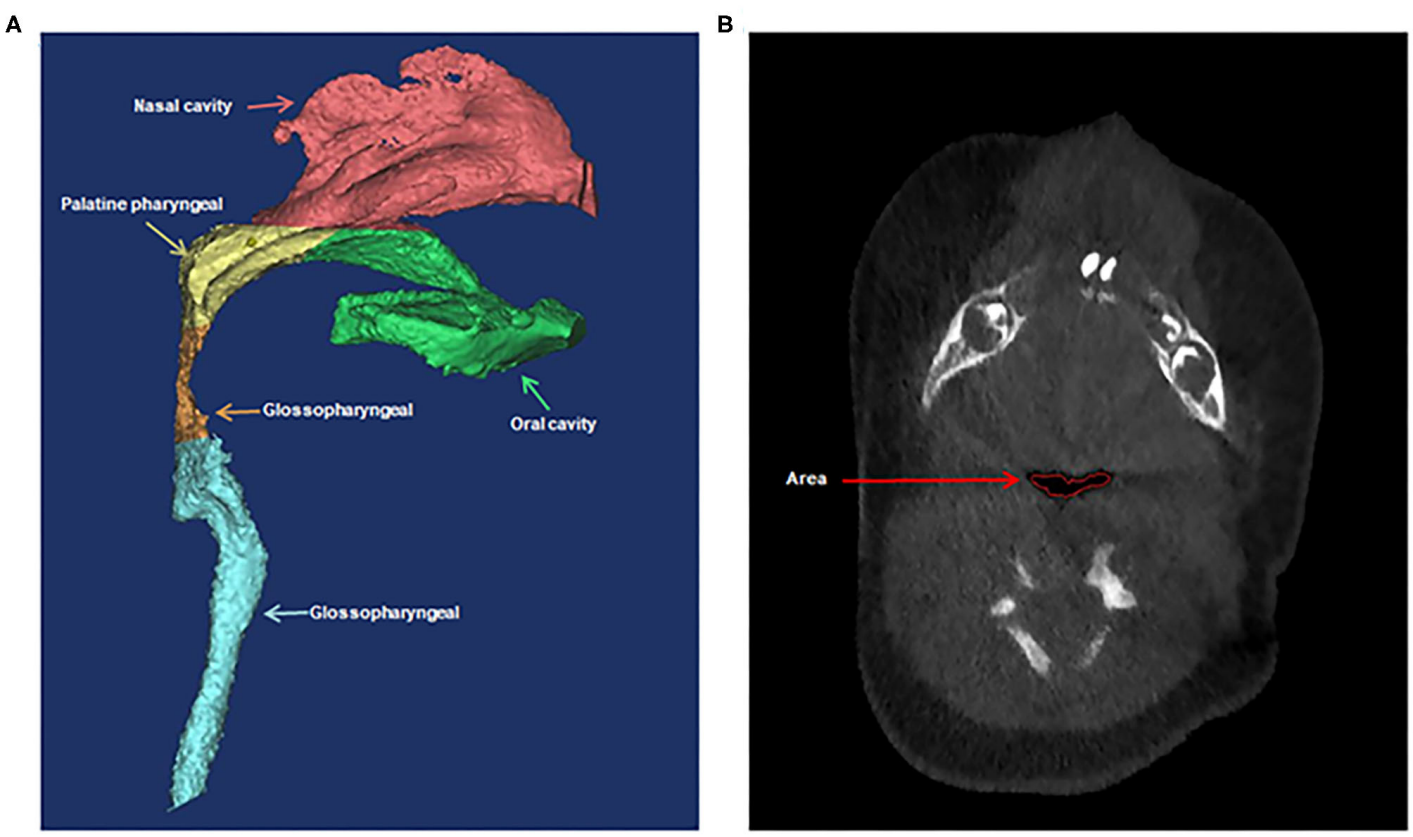
Three-dimensional computed tomographic measurements of the airway volumes **(A)** and area **(B)**. **(A)** The palatine pharyngeal airway from the posterior border of the hard palate to the edge of the soft palate and the glossopharyngeal airway from the tip of soft palate to the upper edge of epiglottis. **(B)** The airway section area at the tip of epiglottis.

Variables that might associate with prolonged mechanical ventilation were analyzed. These variables included type of PRS (priori isolated and non-isolated), sex, age, low birth weight (LBW, defined as <2,500 g at birth), weight, body mass index (BMI) percentile (calculated by plotting BMI on age- and gender-specific growth curve), prematurity (defined as <37 weeks' gestation), preexisting cardiac malformation (defined according to echocardiography), laryngomalacia/tracheomalacia (defined according to results of fiberoptic bronchoscopy), difficult laryngoscopic exposure (defined as glottis exposure of grade III/IV based on laryngoscopy), method of tracheal intubation, preoperative and postoperative pulmonary infection (if chest X-ray showed preoperative radiological infiltration, new or advanced radiological infiltration 48–72 h after tracheal intubation, and one of the following three clinical features and symptoms: temperature >38°C, increased or decreased white blood cell count, and presence of purulent secretion in the respiratory tract), preoperative arterial partial pressure of oxygen and carbon dioxide, duration of the operation and postoperative outcome (including tracheal stenosis, second tracheal intubation, and tracheostomy). Cone-beam CT scans were performed using standard institutional protocols before operation and at the end of distraction. CT scan was performed when the patient was sleeping. If the patients couldn't fall asleep naturally, oral chloral hydrate (0.5 ml/kg) was administered. All images were acquired in the left-lateral position at slice thickness between 0.625 and 1.25 mm. Axial images were reformatted parallel to the Frankfort horizontal plane and sagittal images were subsequently generated. All CT reconstruction and analyses were carried out using MIMICS 17.0 image processing software (Materialize NV, Leuven, Belgium). Airway volumes were calculated based on the axial images using region of interest (ROI) analysis set at a threshold for air density using the volume calculator. Volumes occupied by the radio-opaque border of an artificial airway were excluded from the palatine pharyngeal volume and glossopharyngeal volume. Craniocaudal lengths were calculated based on the reconstructed sagittal images. Preoperative and postoperative non-contrast CT of the maxillofacial skeleton was performed to assess mandibular morphology and airway conditions based on the height of the mandible, the length of the mandibular ramus, the gonial angle, the inferior pogonial angle, the airway section area at the tip of epiglottis, and the palatine pharyngeal (from the posterior border of the hard palate to the edge of the soft palate) and glossopharyngeal (from the tip of the soft palate to the upper edge of epiglottis) airway volumes. All of these measurements based on CT are shown in Figures 1, 2.

### Patient Management Protocol

Sedation and mechanical ventilation weaning followed the standardized protocols: while the patients were mechanically ventilated at ICU, primary sedative agents were fentanyl (2 mcg/kg/h) and midazolam (0.1 mg/kg/h) to maintain the Ramsay Sedation Score at 3~4. Midazolam and fentanyl infusions were discontinued at least 3 h prior to planned extubation. Steroids were used before extubation. Criteria to initiate weaning from the ventilator included pharyngeal reflex and spontaneous breath recovery, hemodynamic stability, postoperative distraction of at least 5 mm and 3 days, and respiratory parameters of FiO_2_ <40%, PaO_2_ > 60 mmHg, PaCO_2_ <50 mmHg, SpO_2_ > 95%, positive end-expiratory pressure <5cm H_2_O.

### Statistical Analysis

Statistical analysis was performed using SPSS Statistics version 22.0 (IBM Co., Armonk, NY, USA) to identify variables which are associated with prolonged mechanical ventilation. Continuous variables were expressed as the median and range, and were analyzed using the Shapiro-Wilk normality test. Variables with normal distribution were analyzed using the *t*-test; and those with abnormal distribution were analyzed using the non-parametric Wilcoxon rank sum test. Categorical variables were expressed as frequencies (percentages) and were analyzed using Pearson's chi-square test. The ordinal variable BMI percentile was analyzed using the Wilcoxon rank-sum test. Significant variables identified through the univariate analysis were included in the multivariate logistic regression model to identify independent variables. *P* < 0.05 was considered statistically significant.

## Results

**Figure 3:**
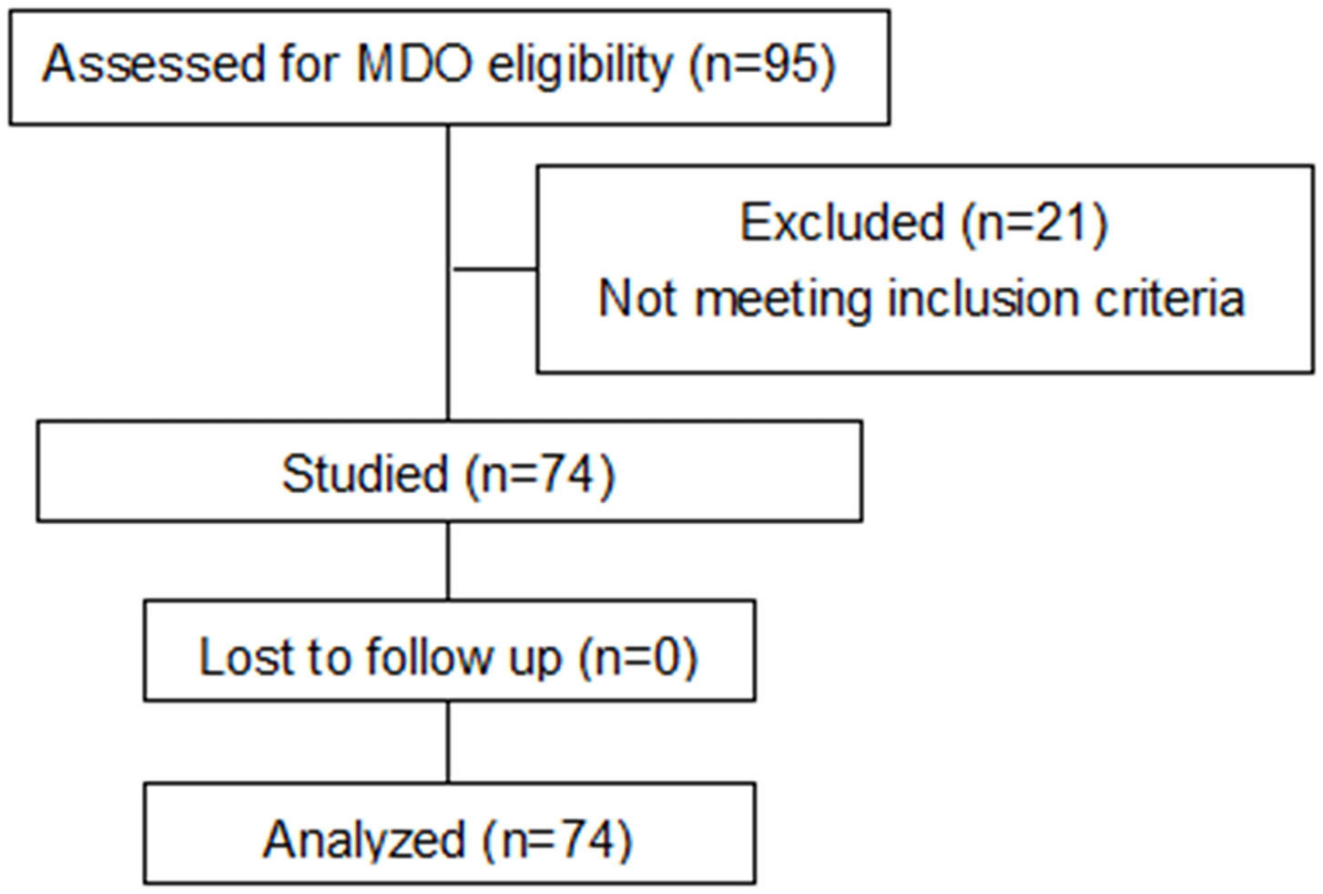
Consort flow diagram following the recruitment of 95 patients.

In this cohort, ~20% of children with PRS ultimately required a surgical intervention. Between November 2016 and November 2018, 95 patients with PRS underwent MDO at our Center out of a total of 475 children diagnosed PRS at the center during the period. However, only 74 fulfilled our inclusion and exclusion criteria for this study and 21 were excluded because of incomplete CT data (Figure 3). Of the 74 infants, 26 (35.1%) underwent prolonged mechanical ventilation, 48 (64.9%) did not. An unexpected detachment of tracheal intubation occurred in one patient 2 days post-surgery, and re-intubation could not be performed due to his respiratory conditions. Four patients required a second intubation, one received tracheostomy due to life-threatening upper airway obstruction, and two patients developed tracheal stenosis. Three patients needed continuous positive airway pressure after extubation. Of the 74 patients, with the median age at the date of surgery was 45.5 days, and the median weight at the date of surgery was 3.4 kg. Fourteen patients had non-isolated PRS, and 65 patients had cardiac malformation, including patent ductus arteriosus, patent foramen ovale, atrial septal defect and ventricular septal defect. All demographic data, the results of the variables between prolonged mechanical ventilation group and control group are presented in Table 1. The age was significantly younger, the preoperative gonial angle was smaller, and the rate of postoperative pulmonary infection was higher in patients with prolonged mechanical ventilation than in those without it (all *P* < 0.05). These variables were then included in a multivariate logistic regression model, and the multivariate analysis revealed that preoperative gonial angle (OR 0.879, 95% CI (0.788–0.981), *P* = 0.021,) and postoperative pulmonary infection [OR 2.851, 95% CI (1.077–8.076), *P* = 0.049] were independent risk factors of prolonged mechanical ventilation (Table 2).

**Table 1:** Demographic characteristics of the 74 patients with Pierre Robin sequence who underwent mandibular distraction osteogenesis.

**Variable**	**Total**	**Prolonged mechanical ventilation**		**Chi-square/u value**	***P*-value**
		**With**	**Without**		
Total (cases)	74	26	48		
Sex [cases (%)]				1.167	0.280
Male	43 (58.1)	15 (57.7)	28 (58.3)		
Female	31 (41.9)	11 (42.3)	20 (41.7)		
Median age at the date of surgery (days)	45.5 (range, 10–207)	33.0 (range, 10–207)	56.0 (range, 15–193)	2.250	0.027
Low birth weight [cases (%)]	15(20.3)	7(46.7)	8(53.3)	1.098	0.295
Median weight at the date of surgery (kg)	3.4 (range, 2.4–6.8)	3.3 (range, 2.4–6.8)	3.5 (range, 2.6–6.0)	0.472	0.319
Body Mass Index Percentile at the date of surgery [cases (%)]				1.040	0.299
The 3th percentile	9 (12.2)	4 (15.4)	5 (10.8)		
The 5th percentile	6 (8.1)	4 (15.4)	2 (4.2)		
The 15th percentile	26 (35.1)	7 (26.9)	19 (39.6)		
The 50th percentile	17 (23.0)	7 (26.9)	10 (20.8)		
The 85th percentile	8 (10.8)	2 (7.7)	6 (12.5)		
The 97th percentile	8 (10.8)	2 (7.7)	6 (12.5)		
Prematurity [cases (%)]	11 (14.8)	3 (11.5)	8 (16.7)	0.062	0.803
Preoperative pulmonary infection [cases (%)]	19 (25.7)	6 (23.1)	13 (27.1)	0.107	0.744
Postoperative pulmonary infection [cases (%)]	41 (55.4)	19 (73.1)	22 (45.8)	5.066	0.024
Laryngomalacia/tracheomalacia [cases (%)]	27 (36.5)	13 (35.3)	14 (37.5)	0.039	0.844
Difficult laryngoscopic exposure [cases (%)]	49 (66.2)	16 (61.5)	33 (68.8)	0.392	0.531
Laryngoscope [cases (%)]	10	2 (7.7)	8 (16.7)	0.521	0.470
Arterial partial pressure of oxygen (kPa)	10.8 (range, 4.0–28.8)	11.2 (range, 5.1–24.8)	10.0 (range, 4.0–28.8)	0.163	0.870
Arterial partial pressure of carbon dioxide (kPa)	5.1 (range, 3.3–9.4)	4.9 (range, 3.6–8.7)	5.4 (range, 3.3–9.4)	1.775	0.076
Duration of the operation (min)	225 (range, 140–505)	225 (range, 150–350)	225 (range, 140–505)	0.109	0.913
Median the height of the mandible (mm)					
Before surgery	15.46 (range, 9.49–21.98)	14.55 (range, 9.49–21.98)	15.72 (range, 11.96–21.37)	1.740	0.086
After surgery	19.96 (range, 12.46–27.00)	19.96 (range, 12.74–25.50)	19.96 (range,12.46–27.00)	1.482	0.073
Median the length of the mandibular ramus (mm)					
Before surgery	40.56 (range, 26.88–51.74)	39.46 (range, 26.88–51.74)	40.75 (range,35.78–49.16)	1.066	0.290
After surgery	56.99 (range, 40.89–64.75)	55.46 (range, 40.89–61.77	57.38 (range, 52.58–64.75)	1.626	0.057
Median the gonial angle					
Before surgery	133.56 (range, 120.44–161.80)	131.21 (range,120.44–142.69)	135.29 (range,122.14-161.80)	2.968	0.002
After surgery	141.61 (range,108.54-161.40)	139.73 (range,108.54–158.53)	142.20 (range,110.38–161.40)	0.597	0.278
Median the inferior pogonial angle					
Before surgery	87.88 (range, 62.92–104.98)	87.88 (range, 62.92–104.98)	88.51 (range, 70.22–103.50)	0.248	0.805
After surgery	72.93 (range, 56.36–96.55)	71.88 (range, 56.36–96.55)	74.42 (range, 59.63–89.40	0.238	0.407
Median airway section area (mm^2^)					
Before surgery	33.00 (range, 0.00–78.14)	29.68 (range, 17.69–78.14)	34.14 (range, 0.00–75.87)	0.968	0.169
After surgery	55.50 (range, 21.93–193.88)	59.10 (range, 34.76–175.80)	47.80 (range, 21.93–193.88)	1.345	0.097
Median palatine pharyngeal volume (mm^3^)					
Before surgery	617.58 (range, 0.00–2010.78)	678.87 (range, 208.60–1495.23)	574.21 (range, 0.00–2010.78)	0.927	0.179
After surgery	1593.31 (range, 237.84–4903.59)	2055.81 (range, 506.19–4903.59)	1503.16 (range, 237.84–4348.60)	1.409	0.086
Median glossopharyngeal volume (mm^3^)					
Before surgery	360.26 (range, 0.00–1086.54)	294.18 (range, 0.00–948.45	369.44 (range, 0.00–1086.54	0.125	0.451
After surgery	654.14 (range, 174.18–1438.09)	637.96 (range, 174.34–1379.44)	658.82 (range, 174.18–1438.09)	0.788	0.220

*Prematurity, 37 weeks gestation; low birth weight, <2,500 g at birth*.

**Table 2:** Multivariate logistic regression analysis of variables associated with prolonged mechanical ventilation.

**Variable**	**B**	**S.E**.	**Wald**	**P**	**OR**	**95% CI**
Age	−0.013	0.008	2.641	0.100	0.987	0.972–1.002
The gonial angle	−0.129	0.056	5.338	**0.021**	0.879	0.788–0.981
Postoperative pulmonary infection	1.048	0.531	3.895	**0.049**	2.851	1.077–8.076

*Preoperative the gonial angle and postoperative pulmonary infection were independent risk factors of prolonged mechanical ventilation (P < 0.05). Bold values meant P < 0.05*.

## Discussion

In the present study, we demonstrated that postoperative pulmonary infection and preoperative gonial angle are independent risk factors of prolonged mechanical ventilation after MDO in infants with PRS (*P* = 0.049). It seems that the duration of mechanical ventilation after MDO and postoperative pulmonary infection compose a reciprocal causation. Postoperative pulmonary infection, a common cause of prolonged mechanical ventilation after surgery, may delay extubation, thus prolonging hospitalization and increasing medical costs (15). On the other hand, a previous study has shown that mechanical ventilation longer than 4 days was also an independent risk factor of postoperative pulmonary infection, and infants with PRS often required mechanical ventilation for more than 4 days (16). How to break this vicious circle is a challenge in the treatment of PRS. Besides, malnutrition, a common situation in infants with severe PRS mainly caused by upper digestive tract motor dysfunction (17, 18), sleep apnoea (19), persistent airway obstruction (20), are also risk factors of postoperative pulmonary infection. Therefore, it is important to take measures to prevent and treat postoperative pulmonary infection in a timely and effective manner, including nutritional support, immunotherapy, and intensive care.

It is generally believed that difficult intubation before surgery and airway obstruction are two important factors that delay extubation (1, 21). However, we did not observe any significant difference in airway obstruction indicators, such as laryngoscopic exposure, the airway cross-sectional area at the tip of epiglottis, the palatine pharyngeal volume, and the glossopharyngeal volume, between patients with and without prolonged mechanical ventilation. We speculate that preoperative airway obstruction and factors predisposing to difficulties in intubation/extubation might have been gradually alleviated by the extension of the mandible. MDO stretches the tongue attachments to the mandible (genioglossus muscle), and thus the position of the tongue moves anteriorly, relieving the glossoptosis (22, 23). Meanwhile, the rate of difficult airway managements following MDO was reduced from 71 to 8.3% in infants with micrognathia (12). Interestingly, we found that infants with prolonged mechanical ventilation have a smaller gonial angle than those without it, suggesting that these patients have flatter mandibles. Compared with normal infants, infants with PRS were found to have shorter and flatter mandibles (24). It is likely that shorter and flatter mandibles may reduce the effective traction of tongue muscle and cause airway obstruction. In addition, due to the hypoplasia of the mandible, the structures such as the larynx and glottis on the attached tongue is relatively backward, resulting in the retroversion of the tongue and narrow pharyngeal cavity. Infants with smaller gonial angle may need longer mandibular distraction to relieve airway obstruction. In the present study, although the height of the mandible and the length of the mandibular ramus were shorter in patients with prolonged mechanical ventilation than in patients without it, the difference was not significant, probably due to the small sample size. Therefore, further studies with more patients are required to determine the impact of mandibular dimensions on the duration of mechanical ventilation after MDO for infants with PRS.

In addition, children with PRS often show symptoms of obstructive sleep apnea syndrome, such as hypoxia and hypercapnia. In this study, we measured the preoperative arterial blood gas of the children, and found that the mean PO_2_ and PCO_2_ are similar between the two groups, suggesting the degree of preoperative hypoxemia and hypercapnia did not affect postoperative extubation. This might be due to alleviated carbon dioxide retention and hypoxia of airway obstruction and hypoxia after mandibular traction. This is consistent with previously findings (25).

Another interesting finding of the present study is that the patients with prolonged mechanical ventilation are significantly younger (33.0 vs. 56.0 days, *P* = 0.027), although age is not an independent risk factor of prolonged mechanical ventilation as demonstrated in the multivariate regression analysis (*P* = 0.100). In addition, weight and gestational age, which are usually correlated with age, showed no significant difference between patients in the two groups in univariate analysis. Younger patients are more difficult in airway maintenance and clearance with less airway distensibility, fewer collateral airways, higher chest wall compliance, poor airway stability, and lower functional residual capacity (26). These physiological characteristics may lead to difficulty in extubation. Our study found that there was no significant difference in the number of children with a non-isolated PRS or an isolated PRS, laryngo-tracheomalacia and cardiac malformation between the two groups. This might partially be due to the surgeon's choice of patients, because infants with severe airway or cardiac malformations, MDO would not be performed. Therefore, in our study, the proportion of patients have a non-isolated PRS was low. Meanwhile, laryngo-tracheomalacia and cardiac malformation in our patients were not very serious. Laryngo-tracheomalacia was mostly mild to moderate, and cardiac malformation only included patent ductus arteriosus, patent foramen ovale, atrial septal defect, ventricular septal defect, which have less impact on hemodynamics. Therefore, the effect of syndromic PRS, severe laryngo-tracheomalacia and cardiac malformation on delayed extubation needs to be addressed with larger sample sizes.

The present study has several limitations. First, the number of patients with delayed extubation was limited. Further studies with larger sample sizes are needed to validate the conclusions. Second, it was a single-center design that would confine the generalization of our findings. For example, the ventilation weaning protocols and extubation readiness tests can vary among intensive care units. Multicenter studies with standardized protocols are needed to verify our conclusions.

## Conclusion

Infants with PRS who have smaller preoperative gonial angle and postoperative pulmonary infection may be more likely to undergo prolonged mechanical ventilation after MDO. Extubation may be attempted within 3–6 days after MDO to reduce possible complications, such as pulmonary infection.

## Data Availability Statement

The raw data supporting the conclusions of this article will be made available by the authors, without undue reservation.

## Ethics Statement

This study was approved by the Research Ethics Committee of Guangzhou Women and Children's Medical Center. Since the study was retrospective, the Research Ethics Committee of Guangzhou Women and Children's Medical Center agreed to waive patient parental consent to review their medical records. Images relating to participants in the manuscript were obtained with written informed consent from the guardian. The study protocol was complied with the 1975 Declaration of Helsinki. Written informed consentto participate in this study was provided by the participants' legal guardian/next of kin.

## Author Contributions

NZ and YT designed study. ZM and YC assisted data analysis and manuscript drafting. NZ and YX analyzed data and drafted manuscript. All authors read and approved the final manuscript.

## Conflict of Interest

The authors declare that the research was conducted in the absence of any commercial or financial relationships that could be construed as a potential conflict of interest.

## Abbreviations

MDO: mandibular distraction osteogenesis
PRS: Pierre Robin sequence
CPAP: continuous positive airway pressure
NPA: nasopharyngeal airway
LBW: low birth weight
BMI: body mass index
CT: computed tomography
OR: relative odds
CI: confident interval
FiO_2_: fraction of inspired oxygen
PaO_2_: partial pressure of oxygen
PaCO_2_: partial pressure of carbon dioxide
SpO_2_: percutaneous oxygen saturation.
